# An Action for Malpraxis in Belgium

**Published:** 1881-10

**Authors:** 


### An Action for Malpraxis in Belgium.

The *Presse Medicale Belge* (April 24) refers to an action brought by the children of a person who had died after the injection of five milligrammes of morphia for the relief of severe pain. The experts called proved that the accusation (brought, it is said, at the instigation of a rival practitioner) was wholly groundless, the individual having in reality died from the natural results of organic disease. The tribunal not only acquitted the accused, but adjudged the persons who brought the accusation to pay 1000 fr. damages to him. If this latter action were followed up in other countries these unfounded accusations would soon diminish—*Med. Times and Gazette, May 7, 1881.*

